# Genomic characterization of the complete terpene synthase gene family from *Cannabis sativa*

**DOI:** 10.1371/journal.pone.0222363

**Published:** 2019-09-12

**Authors:** Keith D. Allen, Kevin McKernan, Christopher Pauli, Jim Roe, Anthony Torres, Reggie Gaudino

**Affiliations:** 1 Steep Hill Labs, Berkeley, California, United States of America; 2 Medicinal Genomics, Woburn, Massachusetts, United States of America; Michigan State University, UNITED STATES

## Abstract

Terpenes are responsible for most or all of the odor and flavor properties of *Cannabis sativa*, and may also impact effects users experience either directly or indirectly. We report the diversity of terpene profiles across samples bound for the Washington dispensary market. The remarkable degree of variation in terpene profiles ultimately results from action of a family of terpene synthase genes, only some of which have been described. Using a recently available genome assembly we describe 55 terpene synthases with genomic context, and tissue specific expression. The family is quite diverse from a protein similarity perspective, and subsets of the family are expressed in all tissues in the plant, including a set of root specific monoterpene synthases that could well have agronomic importance. Ultimately understanding and breeding for specific terpene profiles will require a good understanding of the gene family that underlies it. We intend for this work to serve as a foundation for that.

## Introduction

Most terpenes, particularly the more volatile 10 carbon monoterpenes and 15 carbon sesquiterpenes, are secondary metabolites whose synthesis evolved in response to selection for increased fitness for some ecological niche. Terpenes play central roles in plant communication with the environment, including attracting beneficial organisms, repelling harmful ones, and communication between plants [1]. Monoterpenes and sesquiterpenes are also responsible for most of the odor and flavor properties of *C*. *sativa*, meaning that variation in terpene content is an important differentiator between cultivars. For this reason alone, there has long been interest from breeders in creating cultivars with particular terpene profiles. Further, there is a growing body of preliminary evidence that terpenes play a role in the various effects of *C*. *sativa* on humans, either directly or by modulating the effect of the cannabinoids [2], meaning that medical *C*. *sativa* breeding may include terpene targets. Lastly, decades of research on the role played by terpenes in other plant species suggests that some terpenes may have substantial agronomic impacts [1]. While more than 100 terpenes have been identified in *C*. *sativa* [3], the number routinely observed in flower samples is in the range of 30 to 35, most of which can be identified against available standards.

Consistent with a role in environmental adaptation, terpenes are made by a family of terpene synthases (TPS) that have evolved such that new TPS with differing product profiles can be derived from existing enzymes by changes to just a few amino acids [4]. There is a common “terpenoid synthase fold” [5], but sequence divergence across the family is very high, just staying within the constraints of maintaining an overall fold and basic configuration of the active site. These enzymes are unusual in that they can be promiscuous both in terms of substrate, (with some being shown to use either the ten carbon geranyl diphosphate (GPP) or the 15 carbon farnesyl diphosphate, (FPP)) and product. Many TPS will produce a mixture of different products from a single substrate (see, for example, [6,7]). The central feature of this evolutionary plasticity is that change of just a single amino acid in the active site can lead to a different product profile [4,8–10]. Angiosperms tend to have moderately large families of these enzymes, some apparently from recent duplications, and others quite distant from each other with both divergent and convergent evolution taking place. Generally, product profile of a given enzyme cannot be determined from sequence similarity [11].

A complete understanding of the *C*. *sativa* TPS family will be a valuable resource for efforts to create new varieties. Functional characterization has been completed for 12 *C*. *sativa* monoterpene and sesquiterpene synthases, along with preliminary genomic characterization of dozens more putative genes [7,12]. But without a reasonably complete reference genome, it has not been possible to determine the total size or genomic arrangement of the family. The Jamaican Lion assembly (GenBank accession GCA_003660325.2) has provided contigs long enough to see cluster organization, so we carried out gene discovery in this more complete genome. The assembly we used to the gene discovery work has a total size of 1.06 gb, and an N50 of 2.6 mb. Availability of a large Purple Kush RNA-Seq data set [13] allowed us to refine gene discovery, verify activity, and provide a view of overall gene structure across the entire *C*. *sativa* transcriptome.

We start with a description of trends in terpene profile data collected from flower samples brought to a *C*. *sativa* testing lab, and then proceed to a genomic description of the TPS gene family. Ultimately everything we see in the terpene data has an underlying explanation in the gene family, and complete characterization of this family will enable the next generation of craft and medicinal breeding.

## Materials and methods

### Terpene extraction and GC-MS analysis of C. sativa inflorescence

Terpenoids were isolated from female C. sativa inflorescence and quantitated by Steep Hill Washington using gas chromatography mass spectrometry (GC-MS) measurement. Between 400–600 milligrams of dried homogenized inflorescence was weighed into 15mL polypropylene tubes and extracted in 10mL HPLC grade acetone containing 1,500 μg/mL cyclohexanone as an internal standard (ISTD). Samples were vortexed for 30 seconds and sonicated for 15 minutes, repeated, and then filtered using 0.2 micron PTFE filters and diluted 1:20 with 50 μL of filtered extract and 950 μL of LC-MS grade methanol into 2mL glass autosampler vials. Prepared samples were then analyzed using 1 μL liquid injection into a split/splitless inlet set on split on a GC-MS-QP2010 (70 eV electron impact (EI) ionization, single quadrupole MS, Shimadzu, Kyoto). Gas chromatographic separation was achieved with Helium as a carrier gas on a RESTEK Rxi-5Sil MS: 30m x 0.25mm x 0.25 μm column using a flow rate of 1.2 mL/minute with column oven starting at 100°C with a 4 minute hold, then ramping to 315°C over 16 minutes with a 4 minute final hold. Identification and retention times for specific analytes were determined using purified standard certified reference materials or Q3 scans and the NIST 70 eV EI Mass Spectral Data Base. Selective Ion Monitoring was performed for quantitation and he peak areas of analytes were quantitated relative to the ISTD using external calibration against commercially available terpene standard mixes (Emerald Scientific, San Luis Obispo, California)

### Expression and transcriptome analysis

For expression and transcriptome analysis we used the published Purple Kush (PK) RNA-Seq data set ([13], SRA Accessions SRR352208, SRR352210, SRR352195, SRR352196, SRR352198, SRR352200, SRR35221, SRR352202, SRR352203, SRR352205). This data set comprises more than 600 million reads, and includes six tissue types: roots, stems, vegetative shoots, pre-flowers (i.e. primordia) and flowers in two stages, early flower (flowers with visible stigmas), and mid-flower (flowers with visible, non-withered stigmas and conspicuous trichomes). This data set does not contain replicates, so no statistics was done with the mapping results. The reference genome we used was the Jamaican Lion assembly (GenBank accession GCA_003660325.2). Because we are using rna-SEQ data from one cultivar mapped onto an assembly for a different cultivar, we started with an estimate of genetic distance within the transcribed regions of the genome. PK reads were mapped to the reference genome using Hisat2 version 2.0.6 [14] setting a maximum intron size of 10kB. Output was filtered for map quality = 60 resulting in just less than 400 million uniquely mapped reads. Coverage was estimated from bam files with samtools (samtools.sourceforge.org), and output was filtered for coverage greater than or equal to 5. Next variants were called using the bcftools mpileup and call commands, and output was filtered for quality scores greater than 100 (www.sanger.ac.uk/science/tools/samtools-bcftools-htslib). This gave 294802 called SNPs across a bit more than 80Mb of genomic sequence, for an overall 0.36% variation rate. This was judged to be an acceptible distance, so sorted bam files for the six tissue specific libraries were processed and converted to RPMK values using Ballgown [15]. A high confidence reference guided transcriptome was calculated using StringTie v1.3.4d [16] with the parameters -c 200 -a 15. This requires a minimum depth of 200, and a minimum overlap of 15 bases past the junction to call a splice junction.

### Gene discovery

Gene discovery and refinement were done along much the same lines described in [17]. A protein query set representing TPS families was constructed based around the PFAM PF01397 profile representing a domain that is conserved across all terpene synthases. Full length sequences containing this domain were combined with the full length *C*. *sativa* TPS protein sequences from [7], to yield a query set of 77 proteins that were used in BLAST searches [18]. against the Jamaican Lion genomic contigs. The Jamaican Lion assembly was sequenced with Pacific Biosciences Sequel II platform. 40Kb reads (version 5 and version 6 CLR chemistry) were utilized to seed the FALCON-UNZIP hierarchical assembly process delivering a 665kb N50 assembly with a BUSCO score of 93.6% completeness. Since the submission of this manuscript more details are available in a pre-print described by McKernan et al. (https://osf.io/7d968/). The initial set of raw BLAST hits was used to identify TPS gene containing regions, and for each raw hit the most informative protein query was determined by maximizing total hit length and percent identity. Next these queries were used to estimate gene models using Exonerate operating in exhaustive mode on the gene region. (Exonerate version 2.2.0, [19]). This gave a set of protein similarity based gene models that were often full length, but due to relatively low levels of conservation in the first exon (particularly when a targetting signal is present) some models were missing part of the first coding exon. Where possible gene models were manually adjusted based on the transcriptome data to yield complete gene structures. Assignment of named genes from [7] was not always obvious due to a combination of sequence variability, and possibly also splice variation, but we have tried to stay consistent with that naming scheme.

## Results

### Variation in terpene profile across cultivars

**Fig 1:**
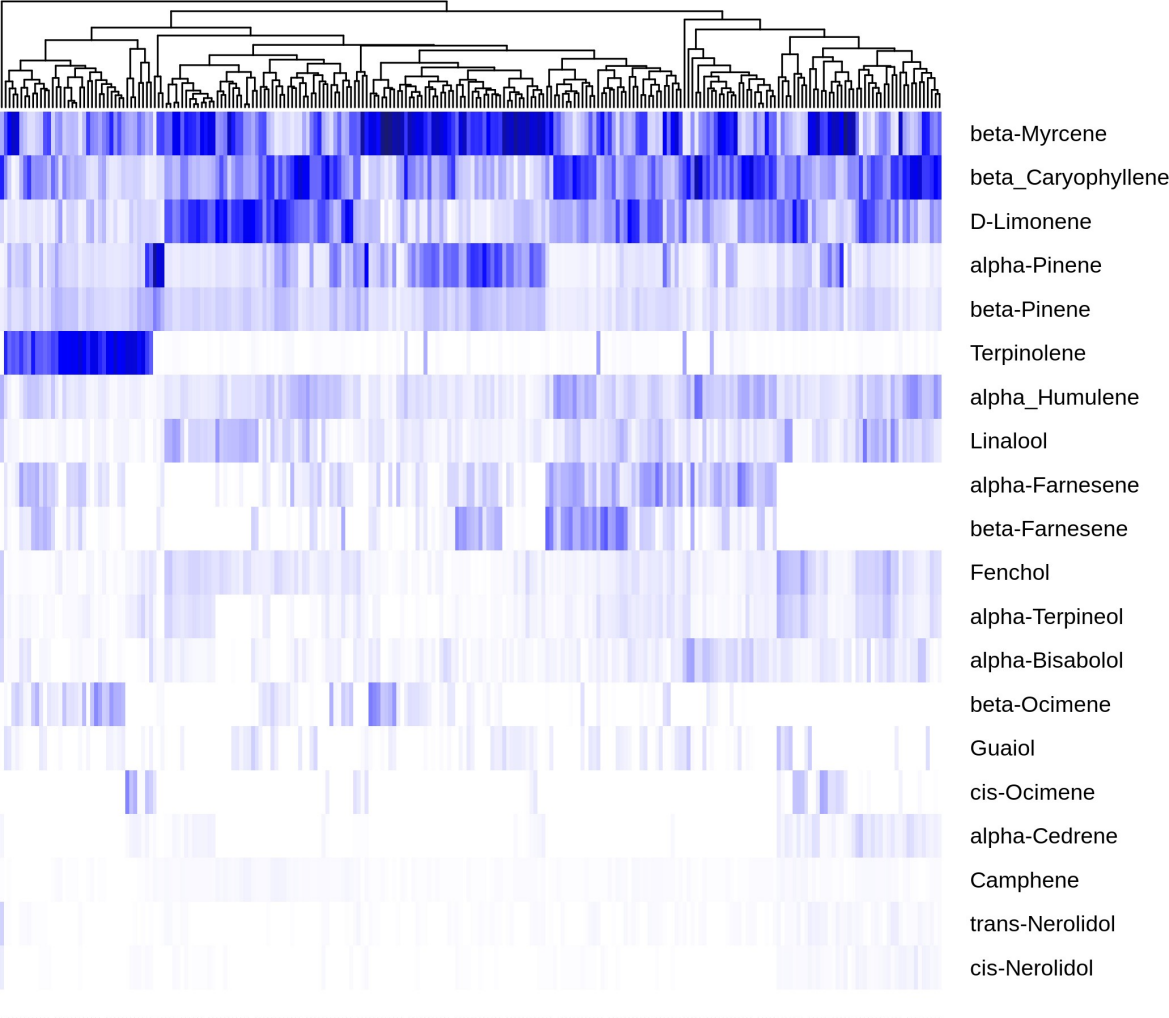
Variation in terpene abundance. Abundance of the twenty most abundant terpenes across 240 cultivars sorted with the most abundant (β-myrcene) at the top. Individual samples were normalized to percent of total terpene present, euclidean distance was calculated with the R dist command prior to clustering with hclust using the “complete” method.

To better understand how terpene content varies across *C*. *sativa* flower bound for the dispensary market in Washington state, GC-MS data for flower samples was filtered for samples with more than 0.5% total terpene to yield 475 samples. These were aggregated by cultivar to give a final data set of 240 cultivars. Note that while this data set represents a broad range of C. sativa samples, Jamaican Lion and Purple Kush (the source, respectively, of the assembly and transcriptome data we are using) are not in this set, so no direct correlations can be made between the oil profile data and the expression results shown below. This data set was used to construct a heat map of the twenty most abundant terpenes (Fig 1). The heatmap has been ordered with the most abundant terpene (β-myrcene) at the top, and hierarchical clustering on the normalized and mean-centered columns (cultivars). This clustering brings together similar profiles and shows some of the patterns in terpene profile.

Starting with overall terpene profile, while typically one or two terpenes dominate a particular sample, the terpene profile in *C*. *sativa* flowers tends to be complex, with an average of 11.2 terpenes present at above 1% of total terpene content, and 5.4 present at above 5% of the total terpenes (S1 Fig). The dominant terpene on average constitutes just 35% of total terpene, and it’s rare, at least in this data set, for a single terpene to constitute more than 50% of total terpene. On average, the top four terpenes comprise just 72% of the total.

The clustering shown in Fig 1 suggests it would be possible to identify chemovars based on terpene profile, just grouping cultivars by the one or two most abundant terpenes. However, differences between cultivar profiles are more complex than this, and a simple classification scheme may miss potentially important variation. One of the driving features of this variability is the dynamic range of individual terpenes. Terpinolene, for example, a cyclic monoterpene with a very distinctive smell, is present at less than 0.01% fresh weight in 86% of the cultivars shown here, but in the remaining 14% of cultivars, it is either the dominant terpene, or close to it. A possible explanation for this would be to posit that there are two enzymes in *C*. *sativa* that produce terpinoline. The first makes terpinolene as it’s primary product, but a second enzyme produces terpinolene as a minor side product, accounting for the background level of terpinolene found in most cultivars. While variability in concentration is most pronounced with terpinolene, it is apparent across the full set of terpenes shown here. In fact, each of the top five terpenes (β-myrcene, α-pinene, D-limonene, β-caryophyllene and terpinolene) vary from being the single most abundant terpene in a cultivar to being a minor component, or in some cases not detectable. The less abundant terpenes, such as the acyclic monoterpenes β- and cis-ocimene (made by TPS6 and TPS13, respectively, [7]), or the acyclic sesquiterpenes α-and β-farnesene, also vary from undetectable levels to being major components in other cultivars.

**Fig 2:**
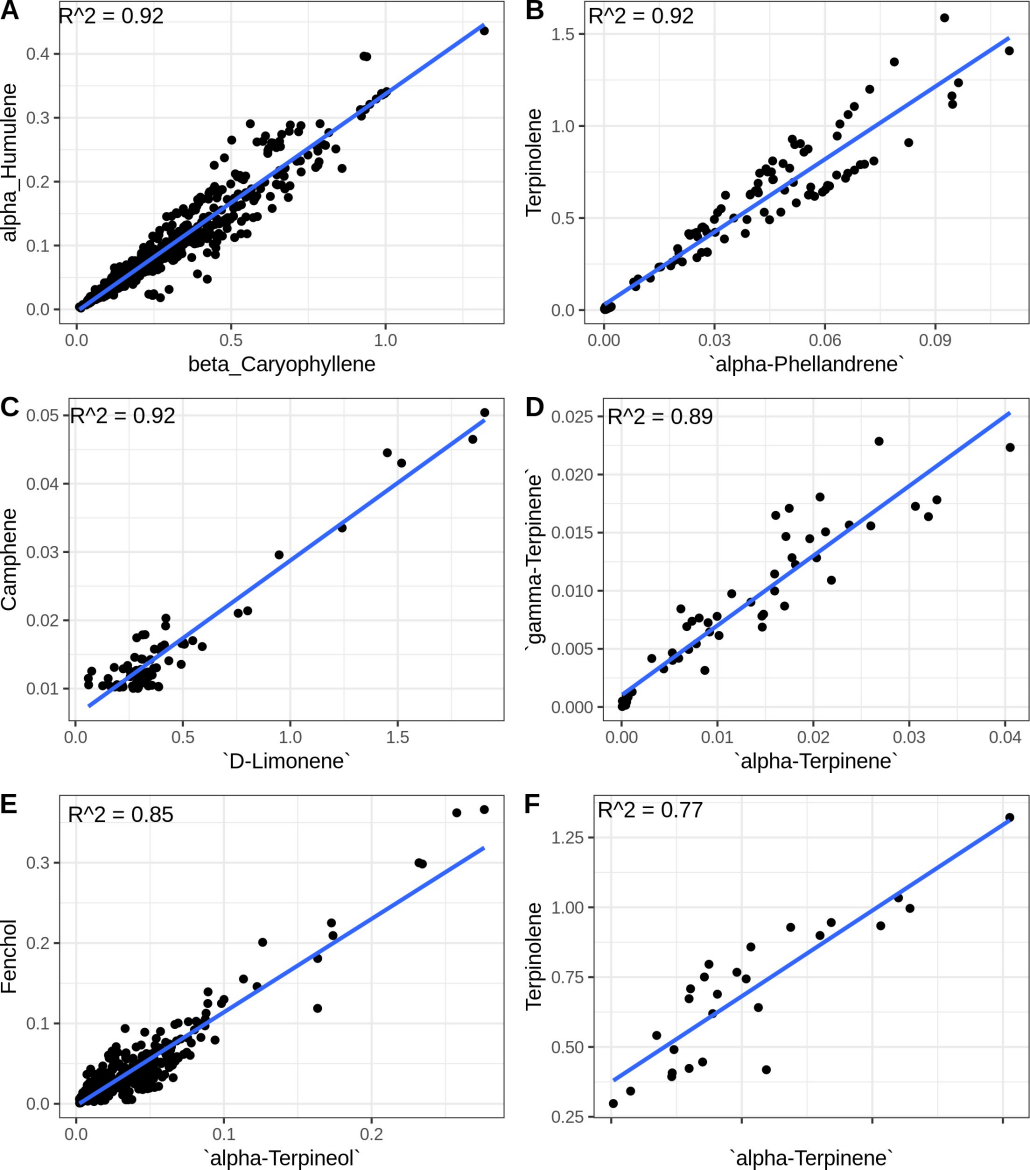
Highly correlated terpenes.Linear relationships between the six most highly correlated compound pairs in this data set. Regression lines were fitted with the R lm command, the R^2 values are shown in the figure, and the F-test p-values for A-E are less than 2.2e-16, while F has a p-value of 3.296e-09.

Another pattern visible in the heatmap is that some terpenes follow each other’s concentration very closely. The most visually obvious example of this is β-caryophyllene and α-humulene, plotted against each other in Fig 2A. Data points are scattered around the regression line consistent with sample variability and perhaps measurement error, but there is a strong linear relationship (R^2 = 0.92). TPS9 has been shown to produce a mixture of β-caryophyllene and α-humulene at a ratio of about 2.5:1 [7]. In our data this ratio is 3.2:1, which is close enough to the *in vitro* result that it could just represent the difference between *in vitro* and *in vivo* environments. Fig 2B–2F show linear relationships between the rest of the top six most highly correlated terpene pairs. The linear relationship between the cyclic monoterpenes α-and γ-terpinene can be explained by the fact that TPS33 has been shown to make these two molecules in approximately equal proportions [7]. These striking linear relationships arise when molecules share a common enzymatic source, but correlation may be degraded when there are multiple enzymatic sources for a pair of molecules.

**Fig 3:**
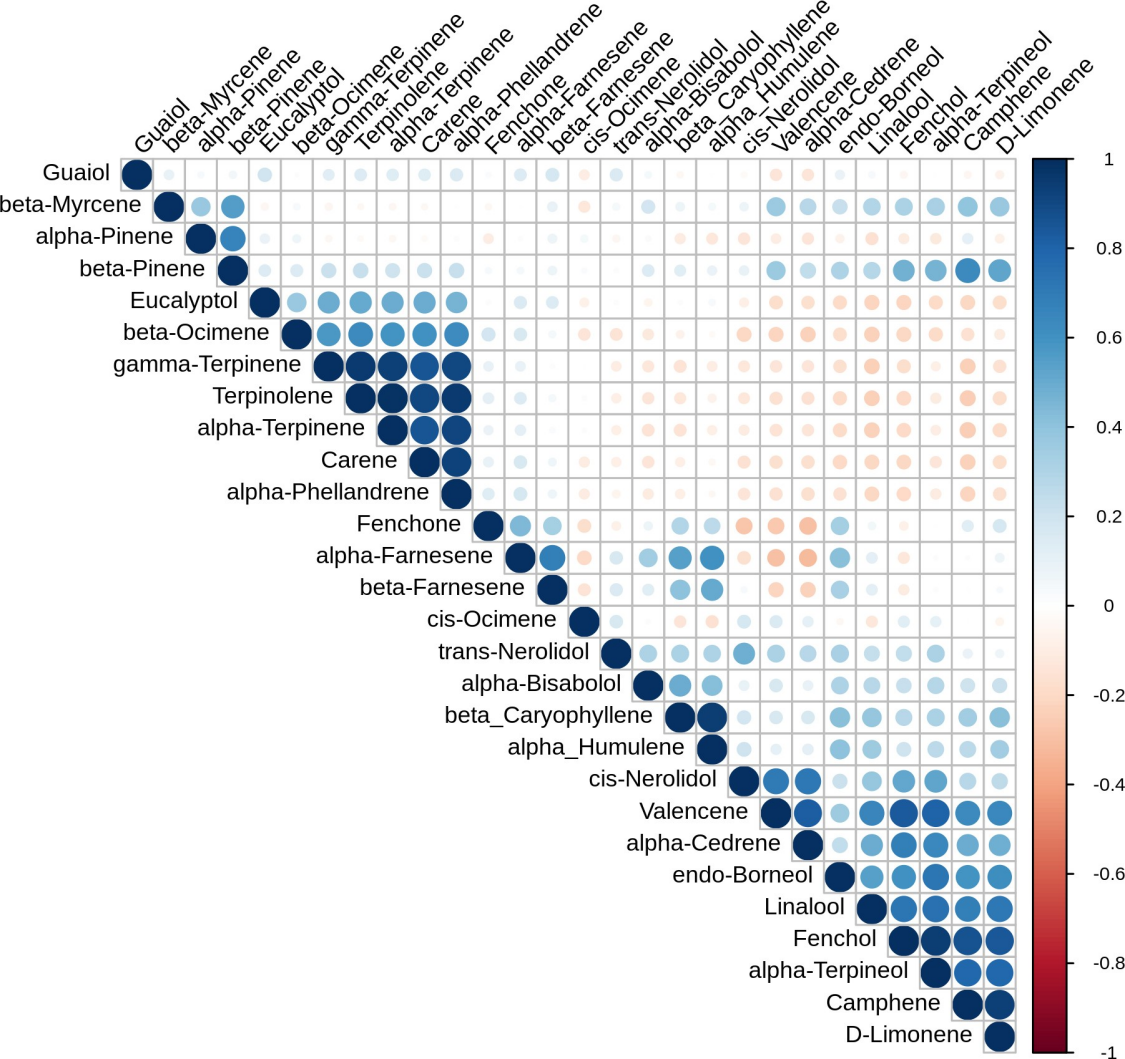
Terpene correlation plot. Visual representation of the correlation matrix, Darker blue color and larger dot size indicate stronger positive correlation, while darker red means stronger negative correlation. Figure generated using the R corrplot package (https://github.com/taiyun/corrplot).

**Fig 4:**
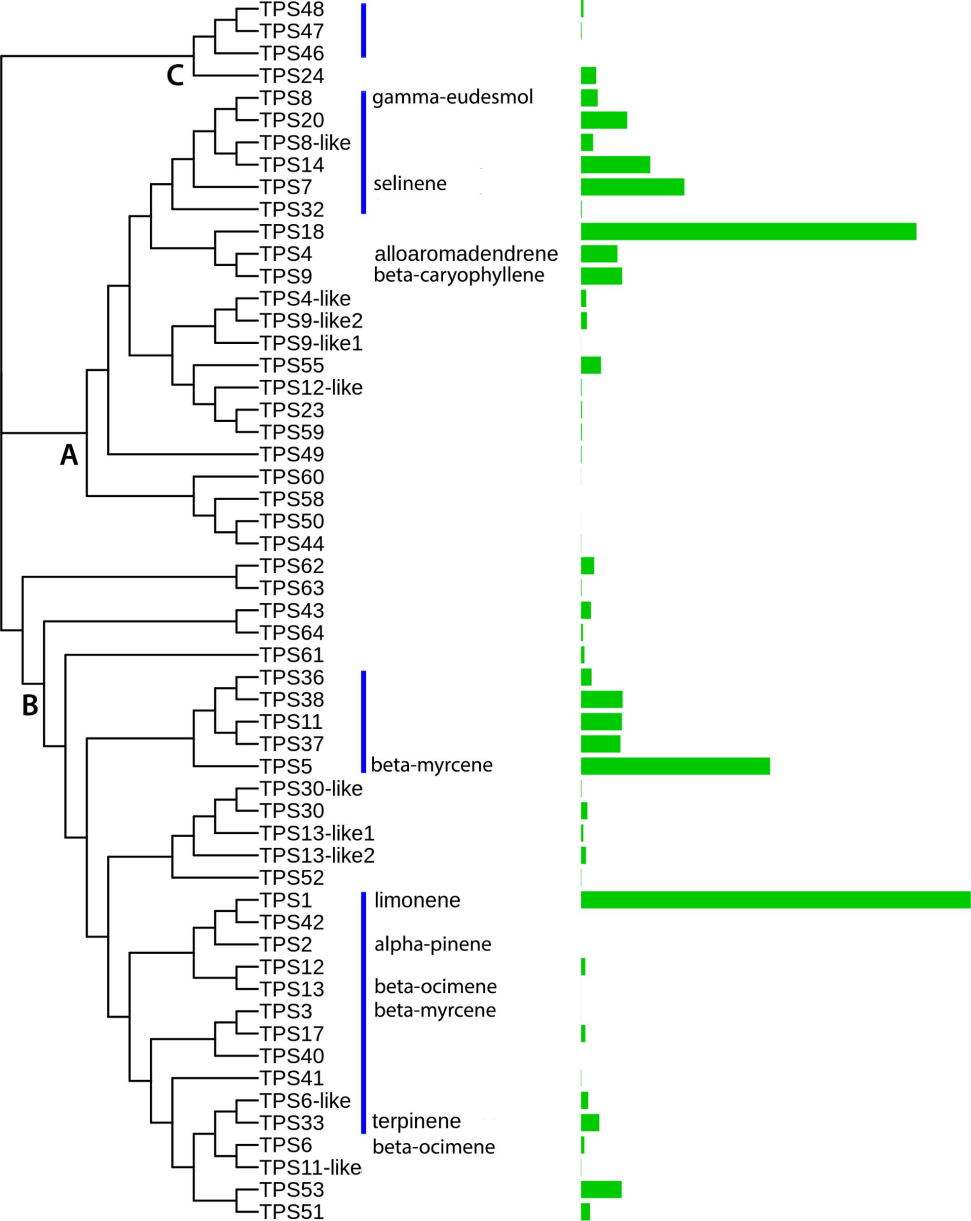
TPS family structure. Neighbor-joining tree calculated for protein sequence of 55 full length TPS genes with ClustalW2. The bar plot on the right shows number of uniquely mapped reads from the combined Purple Kush RNA-Seq data set. TPS families are noted on the tree with A denoting TPS-a sesquiterpene synthases, B TPS-b monoterpene synthases, and C TPS-c diterpene synthases. Blue bars show related genes that exist in clusters on the genomic contigs. Note that TPS7 was not found in the genomic contigs, so the protein sequence was taken from [7].

The larger scale correlation structure of the data set is shown in the correlation plot in Fig 3. Certain correlations like β-caryophyllene and α-humulene stand out, but some examples of lack of correlation are also informative. For example, β-myrcene is fairly independent in the correlation plot, with just a modest correlation to β-pinene (Pearson correlation coefficient = 0.56). So far, two enzymes have been characterized that produce β-myrcene as sole product, TPS3 and TPS30 [7], which would lead you to suspect β-myrcene should be fairly isolated. But TPS5, which is a highly expressed gene in Purple Kush (see Fig 4 below), has been shown to produce approximately equal amounts of β-myrcene and α-pinene [7]. This would seem sufficient to account for the amount of correlation we see. Further, many cyclic monoterpene synthases produce small amounts of β-myrcene as a side product [1]. The *C*. *sativa* β-pinene synthase is as yet unidentified, but it is common for α-and β-pinene synthases in other species to produce some amount of β-myrcene as a by-product [20]. Cis-ocimene is produced as a single product by TPS13 [7], and shows no correlation to other terpenes (Fig 3). The cluster including terpinolene, α-and γ-terpinene, carene and α-phellandrene would be consistent with a yet to be identified terpinolene synthase enzyme that produces this whole group. This fits with current knowledge because all of these molecules could arise from the same carbocation intermediate [6], and are frequently seen as byproducts of synthases that make terpinolene, either as primary product or a byproduct [20,21].

### Terpene synthase family structure and genomic organization

Twelve *C*. *sativa* TPS genes have been functionally characterized and described at the cDNA level, and in the same study about half of the TPS family was detected at least as an initial sequence similarity hit [7]. We conducted exhaustive reference protein-based gene discovery using a Jamaican Lion assembly, with a total size of 1.06 gb and an N50 of 2.6 mb. Our strategy started with blast searches to identify gene containing regions, followed by exonerate searches with the most similar reference TPS sequence as query in order to estimate gene models. This approach yielded 65 candidate genes which were filtered to remove those with multiple frameshifts, or that were otherwise fragmentary. This left 55 complete or essentially complete genes for further analysis. The total number of active TPS genes in *C*. *sativa* may be higher than this since some cultivars may carry active versions of genes we omitted for frameshifts, or because of gaps in the current genome builds, but given the relative completeness of this assembly, we expect this to represent all or most of the family. Fig 4 shows a neighbor joining tree calculated from this set of 55 predicted proteins with ClustalW2 [22], with a bar plot on the right side of the figure showing expression in the Purple Kush RNA-Seq data set [13]. While product profile cannot yet be predicted from protein sequence [6], based on protein similarity we can classify by family, using the characterized TPS1 limonene synthase sequence from *C*. *sativa* [12] and the *Mentha spicata* limonene synthase [23] as exemplars for classification of the TPS-b family, and the characterized TPS9 β-caryophyllene / α-humulene synthase from *C*. *sativa* [7] and the *Abies grandis* γ-humulene synthase as exemplars for identification of TPS-a family members. The exemplar for the TPS-c family was KSG_ARATH, the *Arabidopsis* ent-kaur-16-ene synthase. The TPS-a family is primarily responsible for sesquiterpene synthesis, TPS-b accounts for monoterpene synthesis and TPS-c accounts for diterpene synthesis [10]. These categories are labelled in the tree. The C. sativa TPS family breaks down into 26 TPS-b monoterpene synthases, 21 TPS-a sesquiterpene synthases and four TPS-c diterpene synthases. To get an idea of the sequence diversity of this family, the complete set of pairwise comparisons of the protein sequences was carried out with BLASTP to create a distance matrix. This family is extremely diverse with just 2% of pairwise comparisons having greater than 90% amino acid identity, and the median pairwise identity at just 42%. 54 of these genes had detectable transcripts in the Purple Kush RNA-Seq data set. Total expression in Purple Kush is dominated by just a few genes TPS1, TPS18 and TPS5, but there are 16 genes each accounting for at least 1% of total transcription, and 23 accounting for at least 0.5% of total TPS gene expression. While we lack detailed terpene profile data for Purple Kush, a pattern of expression like this, where a small number of synthases dominate, but the total set of expressed synthases is reasonably large would fit well with the oil profile data we report for dispensary bound samples.

About half of the identified genes are in genomic arrays of various sizes, primarily containing closely related genes. These arrays are shown as blue bars on the dendrogram in Fig 4. The largest TPS cluster contains 11 TPS-b genes, including characterized genes TPS1, TPS2, TPS3 and TPS33, spread over about a megabase, with sections of intervening unrelated genes. This would appear to be the oldest array, as on average these genes share just 66% identity, but there are three pairs of genes in this array that are nearly identical, suggesting more recent local duplications.

**Fig 5:**
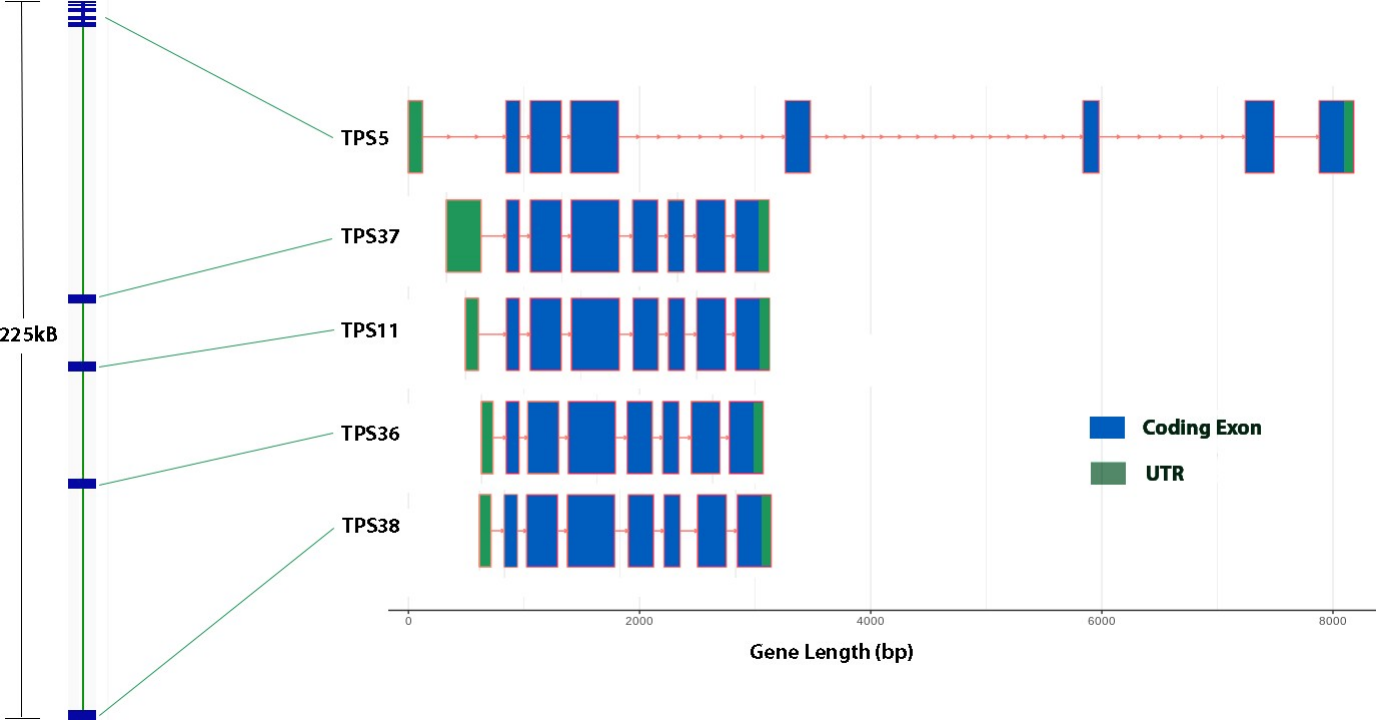
TPS5/TPS11 monoterpene synthase cluster. On the left is the overall organization of this 225kB long cluster, with detailed exon structure on the right.

**Fig 6:**
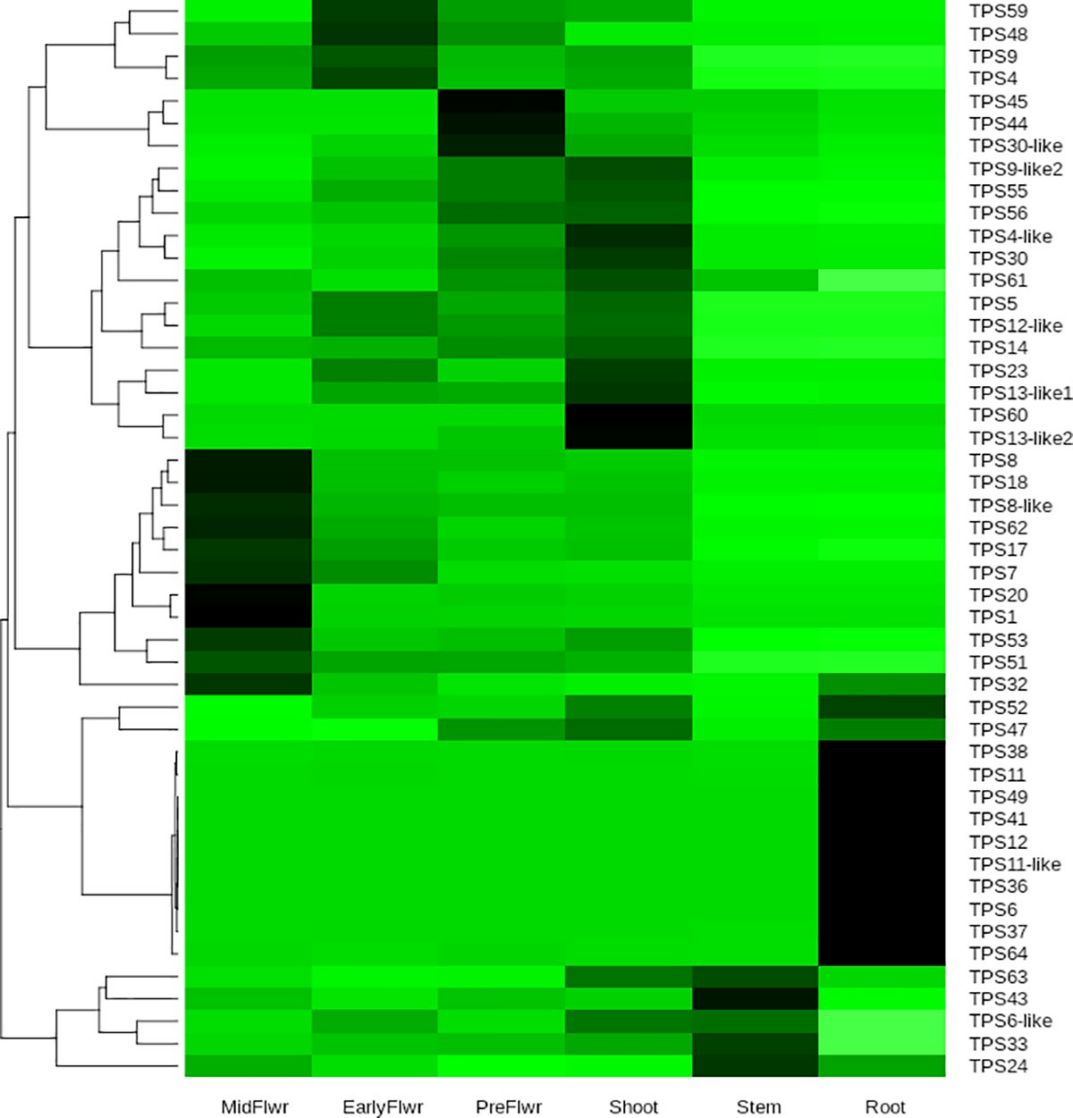
Tissue specific gene expression. Heatmap of terpene synthase gene expression across the six tissues of the Purple Kush RNA-Seq data set [13] Each gene (row) normalized to percent of total expression for each gene (summed across all tissue types). Darker color means higher percentage of total expression for a given gene.

There are two arrays of five genes each, one containing TPS-a genes, and the other TPS-b genes. The first of these spans 135kB, and contains four closely related sesquiterpene synthases, sharing an average of 90% amino acid identity, and including TPS8 (the γ-eudesmol, valencene synthase [7]), and a fifth gene, TPS32, that is slightly more distant at around 81% identity. From a sequence similarity standpoint, TPS7 would be expected to be found in this cluster, but no convincing candidate was found in the Jamaican Lion, Pineapple Banana Bubba Kush (GenBank accession GCA_002090435.1) or Cannatonic (GenBank accession GCA_001865755.1) contigs. The TPS7 gene is clearly expressed, however, in Purple Kush (Fig 4), so failure to find a copy is most likely due to the incomplete nature of the genome assemblies. The second of these five gene arrays spans 225kB and includes TPS5, a β-myrcene / α-pinene synthase [7] and, four uncharacterized monoterpene synthases. These four are nearly identical (on average 97% identical at the protein level), and they have very similar gene structure, with seven coding exons separated by unusually compact introns, a 5’ UTR in a separate exon, and 3’ UTR in the last exon (Fig 5). At 2650 bp in length, these are the shortest terpene synthase genes in *C*. *sativa*. Lastly they all show root specific expression in the Purple Kush data set (see below, Fig 6). TPS5, on the other hand, is on average 73% identical to the four root specific synthases, is one of the longer TPS genes at about 8500 bp, including UTRs, and has a very different expression pattern (see below, Fig 6). Interestingly, the first three coding exons are very similar both structurally and in terms of protein sequence across the entire set of five, with the length difference resulting from large additions into the last four introns.

### RNA-Seq expression analysis

To assess tissue specific expression we used the Purple Kush RNA-Seq data [13], comprising samples from six tissues: root, stem, shoot, pre-flower, early flower, and midflower. This sample set contains no biological replicates for the tissue samples, so no statistical analysis can be done here, but large differences in fragment count can still be judged to be biologically significant. As discussed in Methods, sequence divergence between Purple Kush and Jamaican Lion was estimated at 0.36%, which is certainly compatible with accurate fragment alignment. Purple Kush reads were mapped onto a cDNA set representing the 55 predicted TPS genes using HISAT2 [24], and converted to RPMK values with the R ballgown module [14]. A heatmap of the 48 most abundant transcripts is shown in Fig 6.

Looking at total gene expression across the six tissue types, almost 45% of TPS gene expression occurs in the mid flower sample, while about 10% of expression occurs in roots. Transcripts are detected for 54 genes, but the top five TPS1, TPS5, TPS18, TPS7, TPS14 constitute about 70% of total TPS gene expression (this set includes synthases for D-limonene, β-myrcene and γ-eudesmol, [7]). One or two tissues tend to dominate expression for any given TPS, with the top tissue on average accounting for about 60% of total expression. The expression patterns fall into six clades, one for each of the six tissues in this data set, with the root specific set being the largest and having the highest degree of tissue specificity. Of the 23 genes expressed in root, ten show highly root-specific expression, meaning that roots account for greater than 80% of total expression (six of these roots are 100% root specific). TPS6, the β-ocimene synthase is the only functionally characterized synthase in this set, but nine out of the ten are TPS-b genes and thus expected to be monoterpene synthases. The flower specific clade (combining genes most highly expressed in early and mid-flower samples) accounts for most of the total expression, but these genes show less pronounced specificity than the root specific set. 44 genes have at least some expression in mid-flower plus early flower. These are a mix of mono- and sesquiterpene synthases (either demonstrated or predicted based on sequence similarity).

### Gene structure

**Fig 7:**
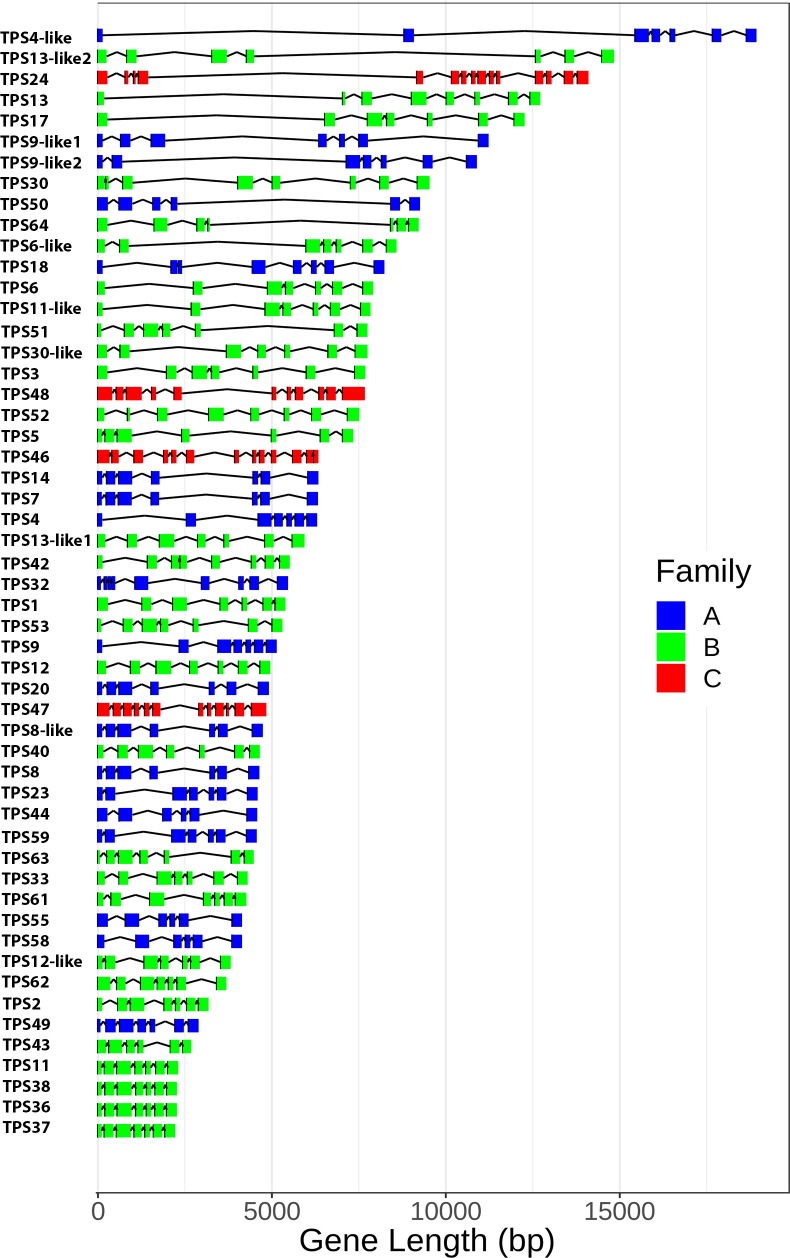
Gene length variation. Gene structure of 55 full length TPS genes. TPS subfamily is indicated by color. A: sesquiterpene synthases, B: monoterpene synthases, C: diterpene synthases.

Gene models for 53 substantially complete terpene synthases (manually curated by combining Exonerate and StringTie outputs) are shown in Fig 7.

In terms of exon structure, the *C*. *sativa* TPS family shows consistent patterns that are highly conserved across angiosperms, gymnosperms, and even bryophytes [1,25] The TPS-a and TPS-b subfamilies, encoding sesquiterpene and monoterpene synthases respectively, tend very strongly to have seven exons, (sometimes containing 5’- or 3’-UTRs in separate exons). Members of the TPS-c family, which is primarily responsible for diterpenes, typically have 15 exons [1], similar to what we observe. While the overall exon/intron structure in the *C*. *sativa* TPS family is conserved, gene sizes vary across a nearly eightfold range from TPS37 at 2223 bp to TPS4-like at almost 19 kbp. Most of this variation comes from intron size, from the very compact TPS37 at 135% of coding sequence length, to TPS4-like at 1150% of coding length. The average intron size for the TPS synthase genes is 984 bp with a median of 673 bp, but many of these genes have one or two exceptionally large introns. 70% of these genes have at least one intron longer than 1000 bp, and 11 have an intron longer than 5000 bp (the longest is in TPS4-like at 8672 bp).

**Fig 8:**
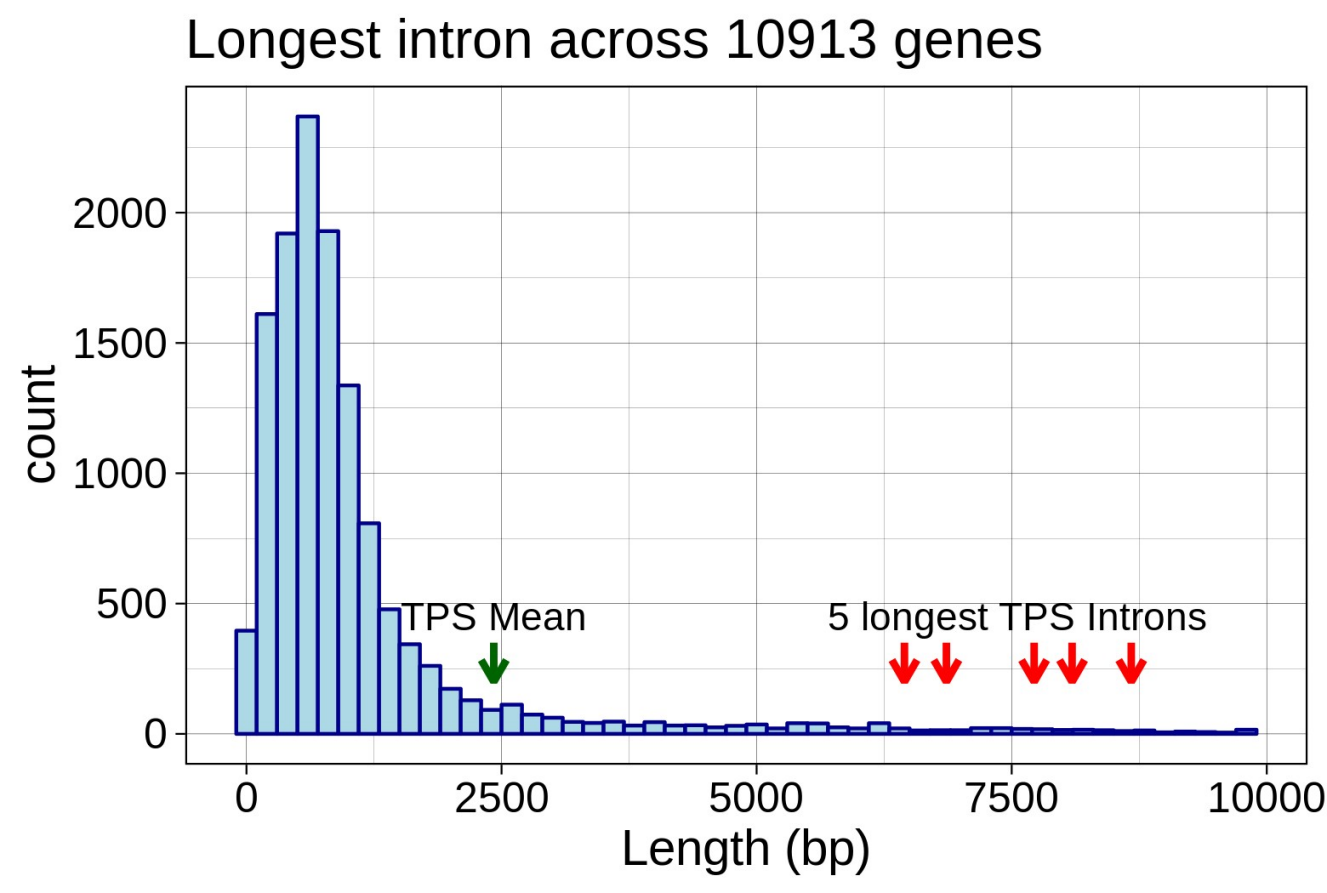
Longest intron size distribution. Distribution of sizes for the longest intron across each of 10,913 multi-exon genes derived from a high confidence transcriptome. The median size, and the sizes from the five largest introns in the TPS family are indicated with arrows.

To investigate whether these introns are unusually long we needed to know more about the distribution of intron sizes in *C*. *sativa*. This is still a largely unannotated genome, so we constructed a high confidence reference guided transcriptome as described in the methods, requiring a read depth of 200 to call an intron junction. This yielded 10,913 transcripts across 9,173 genes with a median coverage of 730 reads. In total, this yields 80,229 introns, with an overall median length of 147 bp, and the most common length (or mode) at 88 bp. This is a highly skewed distribution, with 65% of introns shorter than 250 bp, but less common introns as large as 10 kbp (the maximum length cutoff set for this transcriptome assembly). A recently available assembly of the *C*. *sativa* genome includes annotation using a more comprehensive approach than we’ve used here, [26] and analysis of their annotation shows essentially the same overall intron length distribution. Finally, we compared the longest intron in each TPS gene against the longest introns per gene across the entire transcriptome. The length distribution for the longest introns is shown in Fig 8 (median = 713 bp, mean = 1030 bp), with the longest TPS introns shown as red arrows. The mean length for the longest TPS introns, 2424 bp, is in the top 11% of longest introns across the whole transcriptome, and some TPS introns are among the longest in *C*. *sativa*.

### Active site variation

**Fig 9:**
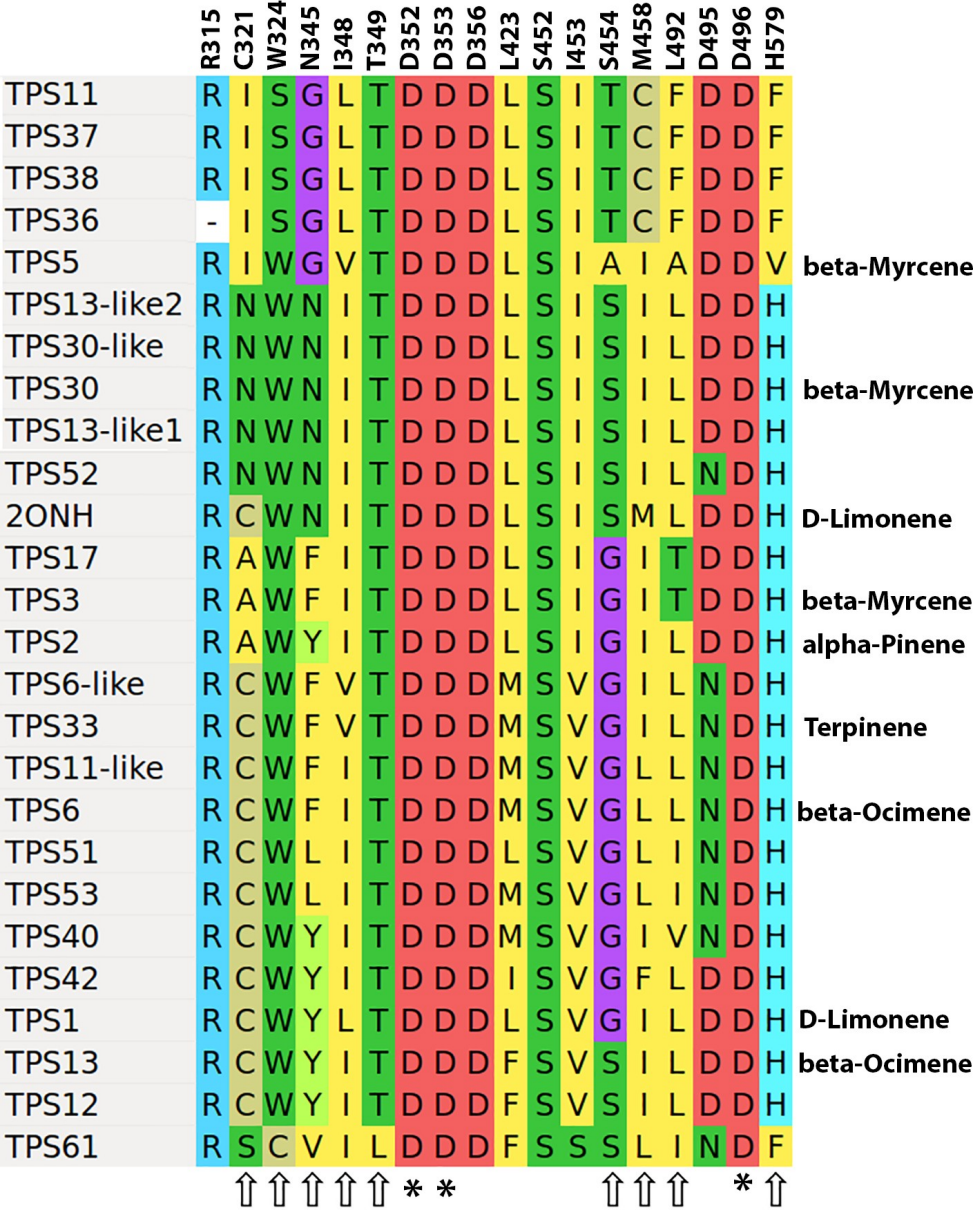
Monoterpene synthase active site residues. Alignment of active site residues for Family B TPS genes. For characterized enzymes the most abundant product with GPP as substrate are shown on the right. Residues indicated with an asterisk (*) are essential for enzyme activity, while those indicated with arrows, if changed will alter the product profile of the enzyme. Amino acid numbering is according to the alignment in Dataset S4 of [9].

Both the monoterpene and sesquiterpene synthase active sites have been extensively studied through crystallographic, mutational, and functional activity assays [6]. For example, using the crystal structure of the *Mentha spicata* D-limonene synthase 2ONH [26], alanine scanning mutagenesis was used to show that only residues within 5 angstroms of the substrate had a strong impact on enzyme output. The 24 monoterpene synthases identified in this study were aligned to the *Mentha spicata* 2ONH sequence, and the aligned active site residues are shown in Fig 9. Both the monoterpene and sesquiterpene synthase active sites contain a set of completely conserved residues that, when mutated, result in complete loss of enzymatic activity [10]. The rest of the active site consists of highly variable positions, or plasticity residues, mutation of which can lead to a change in product profile of the enzyme [4,8,9,27]. These are indicated in Fig 9. Known major products from the published *C*. *sativa* characterization work are shown on the right hand side of the figure [7,12].

**Fig 10:**
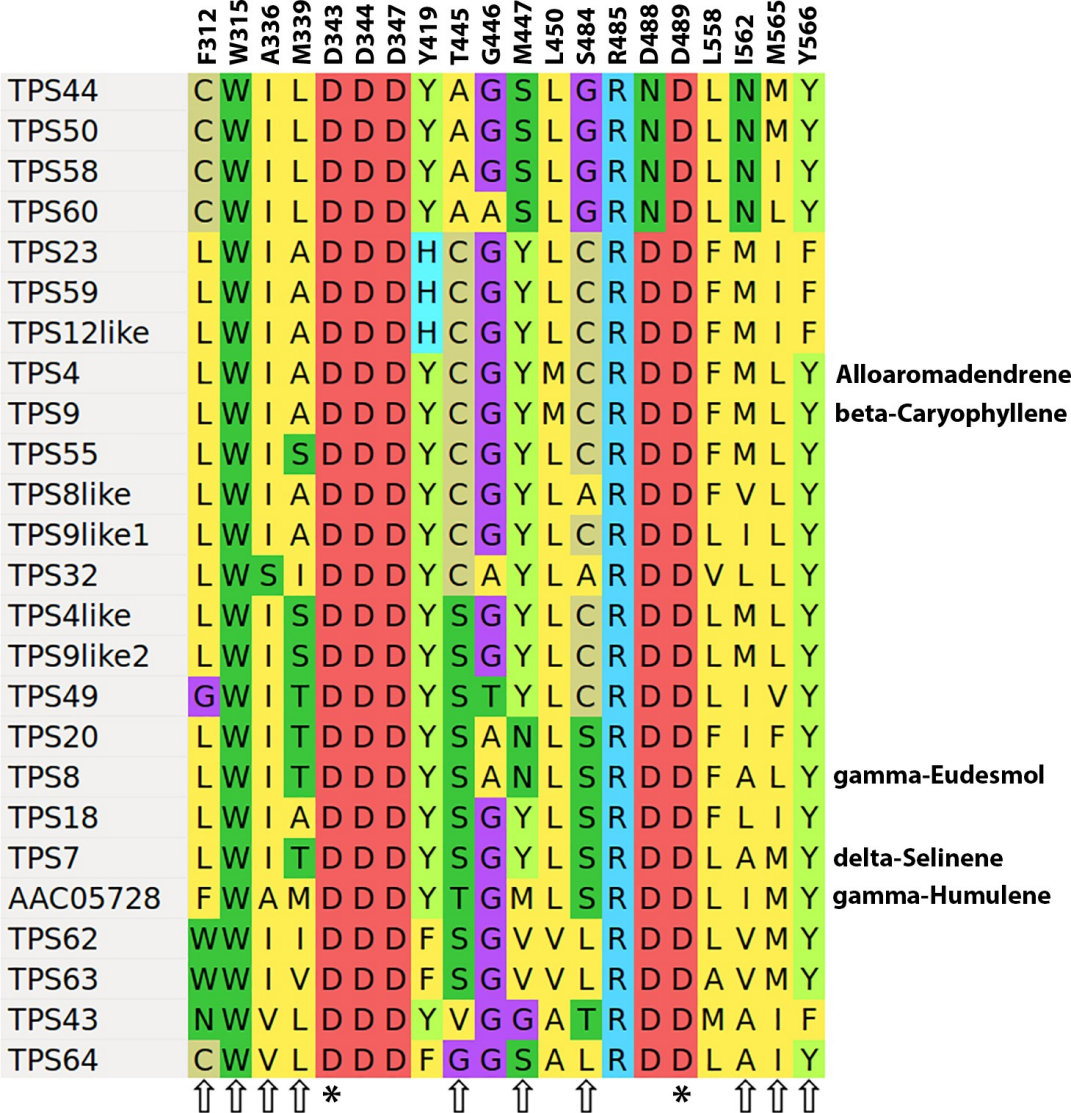
Sesquiterpene synthase active site residues. Alignement of active site residues for Family A TPS genes, aligned to the *Abies grandis* γ-humulene synthase AAC05728. For characterized enzymes the most abundant product with FPP as substrate are shown on the right. Residues indicated with an asterisk (*) are essential for enzyme activity, while those indicated with arrows, if changed will alter the product profile of the enzyme. Residue numbering is taken from supplementary Fig 11 in reference 8.

Fig 10 shows an alignment of the active site residues from 25 sesquiterpene synthases, aligned to the *Abies grandis* γ-humulene synthase (AAC05728), where similar studies, including a crystal structure and mutagenesis experiments have been done [8,9]. There are several sets of two or even three of the *C*. *sativa* synthases that are identical for this set of active site residues, but that doesn’t necessarily mean they make the same products. Although TPS4 and TPS9 have identical active site residues, and are 97% identical at the protein level, TPS4 makes primarily alloaromadendrene with side products including α-humulene, while TPS9 produces β-caryophyllene and α-humulene [7].

## Discussion

The terpene profile in *C*. *sativa* flowers is complex, averaging about eleven terpenes present at above 1% total terpene content, and varies across genotypes. All of this complexity arises from a very diverse family of synthases with at least 55 members. The TPS gene family has been investigated in a number of plant species and diverse repertoires of 40 to 152 TPS genes have been reported [1]. This enzymatic diversity reflects the roles these compounds play in environmental adaptation. Different terpenes, and perhaps combinations of terpenes, can attract or repel various organisms both above and below ground, and individual terpenes can play multiple different roles. For example, β-caryophyllene is a defense against a bacterial pathogen of *Arabidopsis* [28], while in maize, the same molecule is released by roots under attack by western corn rootworm to attract entomopathogenic nematodes, which then attack the root worms [29]. In a citrus hybrid, monoterpene emissions are triggered by insect herbivory, but the mix of terpenes released depends on whether the herbivory is above ground or below ground. In response to below ground attack by root weevil, the mix of monoterpenes, dominated by β-pinene, attracts entomopathogenic nematodes that attack the weevil [30]. Further, terpenes and other volatiles have been shown to define bacterial niches, with bacterial population density varying strongly based on scent emissions [31]. The finding of a root specific expression clade in the *C*. *sativa* TPS family is particularly interesting in light of the role volatile terpenes play in plant interaction with the soil biome. Only one of these genes, TPS6, the β-ocimene synthase, has been functionally characterized, and it is not known what role, if any, these compounds play in *C*. *sativa*. But it could well be that the precise terpene profile emitted by *C*. *sativa* varieties, either above or below ground will have substantial agronomic impacts and could easily lead to future breeding targets.

Purple Kush was not among the cultivars in our oil profile data set, so we cannot make any sort of correlation with this expression data, but Booth and colleagues [7] looked for correlation between expression of characterized TPS genes with metabolite concentration in trichome heads from Finola flowers. The only significant correlation found was between TPS1 and D-limonene. This suggests that TPS1 is the primary source of D-limonene in Finola trichomes, as the presence of an independent synthase would tend to degrade any correlation. Following on this, the lack of correlation between TPS3 and myrcene is expected since there are multiple known enzymatic sources for myrcene already, and this number may go up as the remaining TPS-b family members are characterized. The lack of correlation between TPS2 gene expression and α-pinene is consistent with the presence of more than one α-pinene synthase. It seems likely another will be found because there is no identified β-pinene synthase, and it is common for an enzyme that makes one of the pinene isomers to also make the other, either as a minor by-product or in approximately equal proportions [20,32]. This idea is supported by the correlation between α-and β-pinene we see (Fig 3). The terpinolene synthase has yet to be identified, but if it is found to make α- and γ-terpinene, carene, and α-phellandrene as by-products, it would explain the strong co-linearities we see between these compounds. A number of terpinolene synthases described in other species make some or all of these compounds as well [20,21].

The overall gene structure of the individual TPS genes within each family is remarkably well conserved across the range of higher plants, and even in bryophytes [1], and references therein). This is also observed for the *C*. *sativa* TPS-a and TPS-b families, as well as the four TPS-c family members identified here. *C*. *sativa* TPS-a and TPS-b genes do not vary from 7 coding exons, although UTRs may occur in additional exons. Gene length, on the other hand, varies over a nearly eight-fold range, which does appear to be unusual. Although we are not aware of a systematic study of TPS gene length across species, in the large TPS families in *Eucalyptus grandis* [33], *Vitis vinifera* [24], Tomato [34], and *Arabidopsis* [35] TPS-a and TPS-b gene lengths vary across an approximately 2-fold range. So not only are the *C*. *sativa* TPS introns unusually large for *C*. *sativa*, it would seem that they are also unusually large for TPS-a and TPS-b genes more generally. Introns, of course, may harbor a range of different regulatory elements [36,37], but assessing whether these have any functional significance will require further work.

The oil profile correlation structure shows that there are some limits to what we can breed for in *C*. *sativa*. For example, β-caryophyllene and α-humulene occur at close to a 3:1 ratio, consistent with there being a single source for these molecules, TPS9 [7]. So it seems unlikely we could breed an α-humulene dominant cultivar. Something over half of TPS genes in *C*. *sativa* occur in tandem arrays, in keeping with what has been found in other species [1]. This means that especially with the compact TPS clusters one would expect them to be tightly linked genetically. For example, a breeder aiming to create a cultivar containing a particular variant of the β-myrcene / α-pinene synthase TPS5 will also be bringing in a specific set of variants of four root-specific monoterpene synthases.

But these are small limitations. One thing that stands out looking at terpene distribution is the remarkably wide dynamic range of individual terpenes, suggesting that the spectrum of potential breeding targets, where each represents a unique mixture of terpenes, is enormous. It is our intention that this initial genomic characterization of the gene family serves as the foundation for a road map for the next generation of craft and medicinal *C*. *sativa* breeding.

## Supporting information

S1 Fig Prevalence of terpenes in flower samples. A. Number of terpenes detected above 1% ot total terpene content. B. Number of terpenes found above 5% of total terpene content. Median values are indicated with dashed red lines.
(TIF)

S1 File TPS Transcripts. Predicted transcripts for the genes described in this study (with TPS7 from reference 7 added for completeness sake).
(TXT)

S2 File Terpene profile data. Aggregated oil profile data used in this study.
(TSV)

## References

[1] Chen F, Tholl D, Bohlmann J, Pichersky E. The family of terpene synthases in plants: a mid-size family of genes for specialized metabolism that is highly diversified throughout the kingdom. Plant J. 2011;66(1):212–29. 10.1111/j.1365-313X.2011.04520.x 21443633

[2] Russo EB. Taming THC: potential *Cannabis* synergy and phytocannabinoid-terpenoid entourage effects. Br J Pharmacol. 2011;163(7):1344–64. 10.1111/j.1476-5381.2011.01238.x 21749363 PMC3165946

[3] Brenneisen R. Chemistry and Analysis of Phytocannabinoids and Other *Cannabis* Constituents In: Marijuana and the Cannabinoids. Totowa, NJ: Humana Press; 2007 p. 17–49.

[4] Xu J, Ai Y, Wang J, Xu J, Zhang Y, Yang D. Converting S-limonene synthase to pinene or phellandrene synthases reveals the plasticity of the active site. Phytochemistry. 2017;137:34–41. 10.1016/j.phytochem.2017.02.017 28215610

[5] Lesburg CA, Zhai G, Cane DE, Christianson DW. Crystal structure of pentalenene synthase: mechanistic insights on terpenoid cyclization reactions in biology. Science. 1997;277(5333):1820–4. 10.1126/science.277.5333.1820 9295272

[6] Degenhardt J, Köllner TG, Gershenzon J. Monoterpene and sesquiterpene synthases and the origin of terpene skeletal diversity in plants. Phytochemistry. 2009;70(15–16):1621–37. 10.1016/j.phytochem.2009.07.030 19793600

[7] Booth JK, Page JE, Bohlmann J. Terpene synthases from *Cannabis sativa*. PLoS One. 2017;12(3):e0173911 10.1371/journal.pone.0173911 28355238 PMC5371325

[8] Little DB, Croteau RB. Alteration of product formation by directed mutagenesis and truncation of the multiple-product sesquiterpene synthases δ-selinene synthase and γ-humulene synthase. Arch Biochem Biophys. 2002;402(1):120–35. 10.1016/S0003-9861(02)00068-1 12051690

[9] Yoshikuni Y, Ferrin TE, Keasling JD. Designed divergent evolution of enzyme function. Nature. 2006;440(7087):1078–82. 10.1038/nature04607 16495946

[10] Srividya N, Davis EM, Croteau RB, Lange BM. Functional analysis of (4 *S*)-limonene synthase mutants reveals determinants of catalytic outcome in a model monoterpene synthase. Proc Natl Acad Sci. 2015;112(11):3332–7. 10.1073/pnas.1501203112 25733883 PMC4371936

[11] Bohlmann J, Meyer-Gauen G, Croteau R. Plant terpenoid synthases: molecular biology and phylogenetic analysis. Proc Natl Acad Sci U S A. 1998;95(8):4126–33. 10.1073/pnas.95.8.4126 9539701 PMC22453

[12] Günnewich N, Page JE, Köllner TG, Degenhardt J, Kutchan TM. Functional expression and characterization of trichome-specific (-)-limonene synthase and (+)-α-pinene synthase from *Cannabis sativa*. Nat Prod Commun. 2007;2(3):223–32.

[13] van Bakel H, Stout JM, Cote AG, Tallon CM, Sharpe AG, Hughes TR, et al The draft genome and transcriptome of *Cannibis sativa*. Genome Biol. 2011;12(10):R102 10.1186/gb-2011-12-10-r102 22014239 PMC3359589

[14] Pertea M, Kim D, Pertea GM, Leek JT, Salzberg SL. Transcript-level expression analysis of RNA-seq experiments with HISAT, StringTie and Ballgown. Nat Protoc. 2016;11(9):1650–67. 10.1038/nprot.2016.095 27560171 PMC5032908

[15] Frazee AC, Pertea G, Jaffe AE, Langmead B, Salzberg SL, Leek JT. Flexible isoform-level differential expression analysis with Ballgown. 2014; 10.1101/003665

[16] Pertea M, Pertea GM, Antonescu CM, Chang T-C, Mendell JT, Salzberg SL. StringTie enables improved reconstruction of a transcriptome from RNA-seq reads. Nat Biotechnol. 2015;33(3):290–5. 10.1038/nbt.3122 25690850 PMC4643835

[17] Butler JB, Freeman JS, Potts BM, Vaillancourt RE, Grattapaglia D, Silva-Junior OB, et al Annotation of the *Corymbia* terpene synthase gene family shows broad conservation but dynamic evolution of physical clusters relative to *Eucalyptus*. Heredity (Edinb). 2018;121(1):87–104. 29523839 10.1038/s41437-018-0058-1 PMC5997730

[18] Altschul SF, Madden TL, Schäffer AA, Zhang J, Zhang Z, Miller W, et al Gapped BLAST and PSI-BLAST: a new generation of protein database search programs. Nucleic Acids Res. 1997;25(17):3389–402. 10.1093/nar/25.17.3389 9254694 PMC146917

[19] Slater G, Birney E. Automated generation of heuristics for biological sequence comparison. BMC Bioinformatics. 2005;6(1):31. 15713233 10.1186/1471-2105-6-31 PMC553969

[20] Fähnrich A, Krause K, Piechulla B. Product Variability of the ‘Cineole Cassette’ Monoterpene Synthases of Related *Nicotiana* Species. Mol Plant. 2011;4(6):965–84. 10.1093/mp/ssr021 21527560

[21] Huber DPW, Philippe RN, Godard K-A, Sturrock RN, Bohlmann J. Characterization of four terpene synthase cDNAs from methyl jasmonate-induced Douglas-fir, *Pseudotsuga menziesii*. Phytochemistry. 2005;66(12):1427–39. 10.1016/j.phytochem.2005.04.030 15921711

[22] Thompson JD, Higgins DG, Gibson TJ. CLUSTAL W: improving the sensitivity of progressive multiple sequence alignment through sequence weighting, position-specific gap penalties and weight matrix choice. Nucleic Acids Res. 1994;22(22):4673–80. 10.1093/nar/22.22.4673 7984417 PMC308517

[23] Hyatt DC, Youn B, Zhao Y, Santhamma B, Coates RM, Croteau RB, et al Structure of limonene synthase, a simple model for terpenoid cyclase catalysis. Proc Natl Acad Sci. 2007;104(13):5360–5. 10.1073/pnas.0700915104 17372193 PMC1838495

[24] Kim D, Langmead B, Salzberg SL. HISAT: a fast spliced aligner with low memory requirements. Nat Methods. 2015;12(4):357–60. 10.1038/nmeth.3317 25751142 PMC4655817

[25] Martin DM, Aubourg S, Schouwey MB, Daviet L, Schalk M, Toub O, et al Functional Annotation, Genome Organization and Phylogeny of the Grapevine (*Vitis vinifera*) Terpene Synthase Gene Family Based on Genome Assembly, FLcDNA Cloning, and Enzyme Assays. BMC Plant Biol. 2010;10(1):226. 20964856 10.1186/1471-2229-10-226 PMC3017849

[26] Grassa CJ, Wenger JP, Dabney C, Poplawski SG, Motley ST, Michael TP, et al A complete *Cannabis* chromosome assembly and adaptive admixture for elevated cannabidiol (CBD) content. bioRxiv. 2018;458083.

[27] Greenhagen BT O’Maille PE, Noel JP, Chappell J. Identifying and manipulating structural determinates linking catalytic specificities in terpene synthases. Proc Natl Acad Sci U S A. 2006;103(26):9826–31. 10.1073/pnas.0601605103 16785438 PMC1502538

[28] Huang M, Sanchez-Moreiras AM, Abel C, Sohrabi R, Lee S, Gershenzon J, et al The major volatile organic compound emitted from *Arabidopsis thaliana* flowers, the sesquiterpene (E)-β-caryophyllene, is a defense against a bacterial pathogen. New Phytol. 2012;193(4):997–1008. 10.1111/j.1469-8137.2011.04001.x 22187939

[29] Rasmann S, Köllner TG, Degenhardt J, Hiltpold I, Toepfer S, Kuhlmann U, et al Recruitment of entomopathogenic nematodes by insect-damaged maize roots. Nature. 2005;434(7034):732–7. 10.1038/nature03451 15815622

[30] Ali JG, Alborn HT, Stelinski LL. Constitutive and induced subterranean plant volatiles attract both entomopathogenic and plant parasitic nematodes. J Ecol. 2011;99(1):26–35.

[31] Junker RR, Tholl D. Volatile Organic Compound Mediated Interactions at the Plant-Microbe Interface. J Chem Ecol. 2013;39(7):810–25. 10.1007/s10886-013-0325-9 23881446

[32] Pyun HJ, Wagschal KC, Jung DI, Coates RM, Croteau R. Stereochemistry of the Proton Elimination in the Formation of (+)- and (−)-α-Pinene by Monoterpene Cyclases from Sage (*Salvia officinalis*). Arch Biochem Biophys. 1994;308(2):488–96. 10.1006/abbi.1994.1069 8109979

[33] Külheim C, Padovan A, Hefer C, Krause ST, Köllner TG, Myburg AA, et al The *Eucalyptus* terpene synthase gene family. BMC Genomics. 2015;16(1):450. 26062733 10.1186/s12864-015-1598-x PMC4464248

[34] Falara V, Akhtar TA, Nguyen TTH, Spyropoulou EA, Bleeker PM, Schauvinhold I, et al The Tomato Terpene Synthase Gene Family. 2011; 10.1104/pp.111.179648. PMC3192577 21813655

[35] Aubourg S, Lecharny A, Bohlmann J. Genomic analysis of the terpenoid synthase (AtTPS) gene family of *Arabidopsis thaliana*. Molecular Genetics and Genomics. 2002 8 1;267(6):730–45. 10.1007/s00438-002-0709-y 12207221

[36] Shaul O. How introns enhance gene expression. Int J Biochem Cell Biol. 2017;91(Pt B):145–55. 10.1016/j.biocel.2017.06.016 28673892

[37] Rose AB. Introns as Gene Regulators: A Brick on the Accelerator. Front Genet. 2019;9:672 10.3389/fgene.2018.00672 30792737 PMC6374622

