# Supplementary figures and images for: Genomic characterization of the complete terpene synthase gene family from Cannabis sativa
Source: PLoS One. 2019 Sep 12;14(9):e0222363. doi: 10.1371/journal.pone.0222363 (PMC6742361; doi:10.1371/journal.pone.0222363)

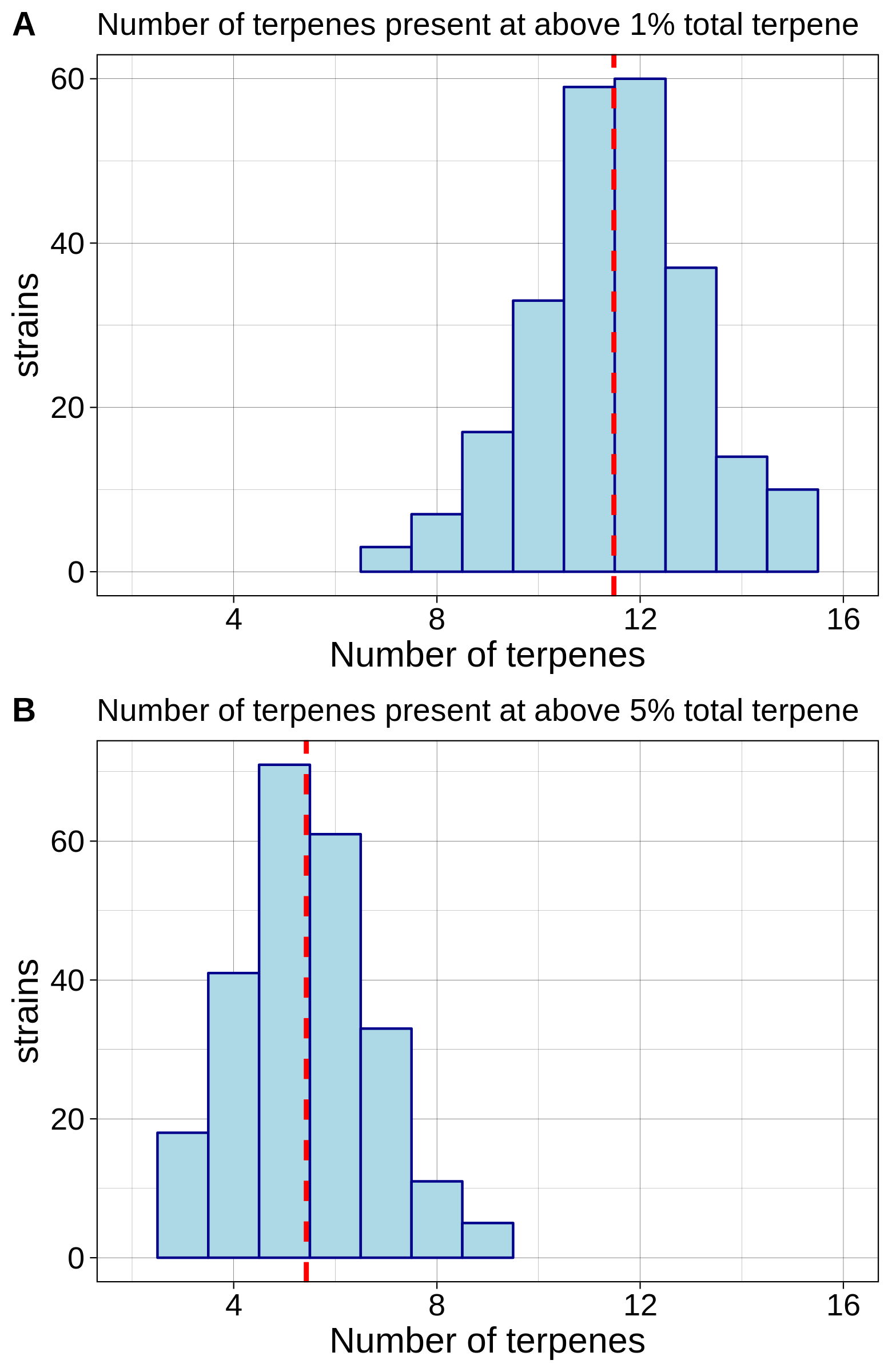

Supplement: S1 Fig — A. Number of terpenes detected above 1% ot total terpene content. B. Number of terpenes found above 5% of total terpene content. Median values are indicated with dashed red lines. (TIF) [file pone.0222363.s001.tif]
